# Risk of infection due to medical interventions via central venous catheters or implantable venous access port systems at the middle port of a three-way cock: luer lock cap vs. luer access split septum system (Q-Syte)

**DOI:** 10.1186/1471-2334-14-41

**Published:** 2014-01-25

**Authors:** Fabian Pohl, Werner Hartmann, Thomas Holzmann, Sandra Gensicke, Oliver Kölbl, Matthias G Hautmann

**Affiliations:** 1 Department of Radiotherapy, University Hospital Regensburg, Regensburg, Germany; 2 Risk Management, Becton Dickinson GmbH, Heidelberg, Germany; 3 Department of Medical Microbiology and Hygiene, University Hospital Regensburg, Regensburg, Germany

**Keywords:** Luer access split septum system, C-port, Central venous catheter, Contamination, Health risks

## Abstract

**Background:**

Many cancer patients receive a central venous catheter or port system prior to therapy to assure correct drug administration. Even appropriate hygienic intervention maintenance carries the risk of contaminating the middle port (C-port) of a three-way cock (TWC), a risk that increases with the number of medical interventions. Because of the complexity of the cleaning procedure with disconnection and reconnection of the standard luer lock cap (referred as “intervention”), we compared luer lock caps with a “closed access system” consisting of a luer access split septum system with regard to process optimization (work simplification, process time), efficiency (costs) and hygiene (patient safety).

**Methods:**

For determination of process optimization the workflow of an intervention according to the usual practice and risks was depicted in a process diagram. For determining the actual process costs, we analyzed use of material and time parameters per intervention and used the process parameters for programming the process into a simulation run (n = 1000) to determine the process costs as well as their differences (ACTUAL vs. NOMINAL) within the framework of a discrete event simulation.

Additionally cultures were carried out at the TWC C-ports to evaluate possible contamination.

**Results:**

With the closed access system, the mean working time of 5.5 minutes could be reduced to 2.97 minutes. The results for average process costs (labour and material costs per use) were 3.92 € for luer lock caps and 2.55 € for the closed access system. The hypothesis test (2-sample *t*-test, CI 0.95, p-value<0.05) confirmed the significance of the result.

In 50 reviewed samples (TWC’s), the contamination rate for the luer lock cap was 8% (4 out of 50 samples were positive), the contamination rate of the 50 samples with the closed access system was 0%.

Possible hygienic risks (related to material, surroundings, staff handling) could be reduced by 65.38%.

**Conclusions:**

In the present research, the closed access system with a divided split septum was superior to conventional luer lock caps. The advantage of the closed access system lies in the simplified handling for staff, which results in a reduced risk of patient infection due to improved clinical hygiene.

## Background

Cytostatics are standard treatment for cancer patients, for example, for patients with esophagus carcinoma [1]. However, cytostatic drug protocols often have complex intravenous infusion schedules with interventions up to 20 times per 24 hours [2]. For this reason, many patients receive a central venous catheter (CVC) or an implantable venous access port system (PORT) prior to therapy to assure correct drug administration and to avoid extravasation.

Depending on the type of cancer, one or more cytostatic drugs are intravenously administered via a CVC or a PORT, as well as parenteral nutrition or other medications, for instance, against nausea and emesis or for stomach protection. In addition, NaCl 0.9% rinsing solutions are used for cleaning catheters of cytostatic drugs and to ensure the patency of the catheter system. All these described administrations are designated as “intervention” in the following text.

**Figure 1:**
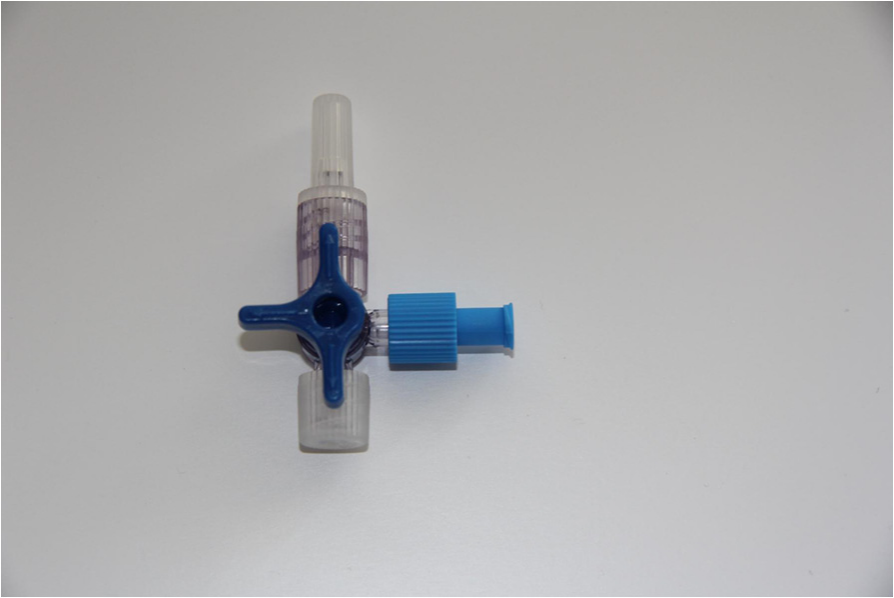
**TWC-luer lock cap (ACTUAL-condition): Blue luer lock cap at C-port of TWC (example of TWC, here “Discofix 3SC®” from B. Braun Melsungen, Germany.** Example of luer lock cap, here “Combi-Stopper, blue®” from Dispomed Witt OHG, Gelnhausen, Germany).

CVC’s or PORT’s are usually supplemented with a large bore extension and a three-way cock (TWC). After each medical intervention or cleaning procedure, the access to the catheter system must be disconnected and then reconnected according to a high hygienic standard by means of a luer lock cap. Figure 1 shows an example of a TWC closed with a blue luer lock cap at the C-port, here the “Discofix 3SC®” from B. Braun Melsungen, Germany, which we use on our ward. The shown luer lock cap is called “Combi-Stopper, blue®” and made by Dispomed Witt OHG, Gelnhausen, Germany. Even appropriate hygienic intervention maintenance carries the risk of contaminating the middle port (C-port) of a TWC, with the possible consequences of bacteremia and sepsis e.g. in patients with Staphylococcus aureus colonization of intravascular catheters [3], a risk that increases with the number of interventions per day [4].

**Figure 2:**
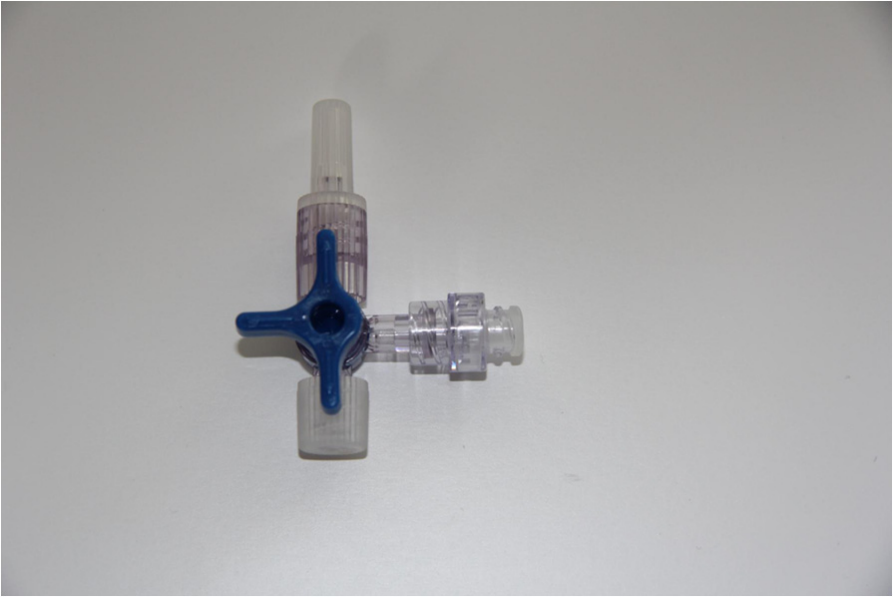
TWC-BD-Q-Syte ® (NOMINAL-condition): Transparent Q-Syte at C-port of TWC.

For some time, different companies offered as an alternative to the luer lock cap for avoiding health risks due to contamination of TWC hubs (C-ports) luer access split septum systems (Figure 2). Luer access split septum systems do not require an additional luer lock cap (Figure 1) because the hub is closed by a silicone split septum. An infusion set can be directly connected to the system after the outer silicone surface of the luer access split septum system has been cleaned with an alcohol pad or an antiseptic. Thus, this device represents a “closed access system” that reduces the risk of contamination of TWC hubs to a minimum.

To avert patient injuries, each medical facility in Germany is obligated by law to quality assurance. An important part is hygienic risk management, and particularly Article 1 of the German Law on the Prevention and Control of Infectious Diseases (German abb.: IfSG) explicitly refers to the direct responsibility of hospitals [5,6].

Because a lower rate of interventions may also be equated with better hygiene, we compared luer lock caps with the new luer access split septum system with regard to the following topics:

1. hygiene (patient safety)

2. process optimization (work simplification, process time)

3. efficiency (costs)

## Methods

Our comparison of the two systems and their application was thereby thematically divided into two phases:

Phase 1/Efficiency and work simplification

Phase 2/Hygiene and patient safety

### Phase 1/Efficiency and work simplification

**Figure 3:**
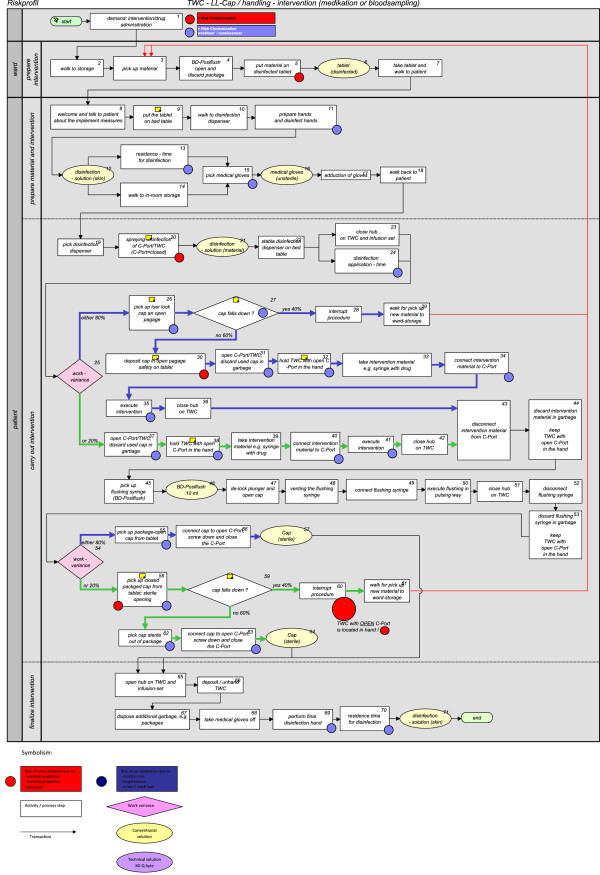
Work process intervention: TWC-luer lock cap (ACTUAL).

The workflow of an intervention according to the usual practice (ACTUAL) was depicted in a process diagram (Figure 3). During each step of the course of the workflow, on-site interviews were conducted with experienced nurses who had worked at our clinic for many years. The individual steps were logged in the process diagram. The steps are marked with numbers in each diagram, e.g.

1. demand: intervention/drug administration

2. walk to storage

3. pick up material

4. BD-Posiflush open and discard package

5. put material on disinfected tablet

6. tablet (disinfected)

7. take tablet and walk to patient

8. welcome and talk to patient about the implement measures

9. put the tablet on bed table

**Figure 4:**
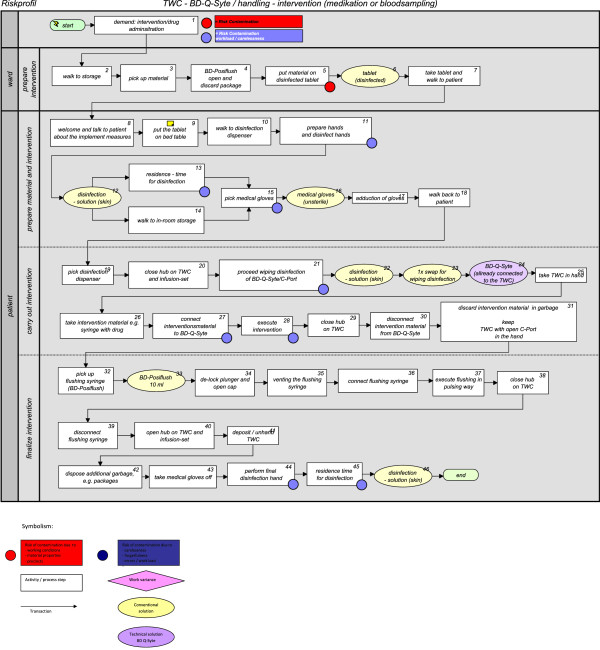
Work process intervention: TWC-BD-Q-Syte ® (NOMINAL).

Time per every activity was measured 5 times, and the material components of the process were recorded per single use. These process parameters were used for programming the process into a simulation run (n = 1000) to determine the process costs per intervention as well as their differences (ACTUAL vs. NOMINAL) within the framework of a discrete event simulation [7]. The contamination risks of the TWC-port (C-port) were recorded in discrete events, whereas the causes of the risks in the work process were marked with a color code. The analysis of a possible NOMINAL sequence (TWC BD Q-Syte®) was similarly edited (Figure 4).

For one-time usable luer lock caps, the recording of the ACTUAL work sequence already showed variances in terms of the preparation and application of the caps with regard to time used or staff handling that contributed to increasing the hygienic risk. Possible work variances were, for example, caps being carried in lab coat pockets, luer lock caps falling to the ground, and contamination of caps during their removal from packaging, even on the seal side (the position of the luer lock caps in the packaging varies, disallowing blind removals).

### Statistical analysis

The average process costs (material and labour) between the luer access split septum system and the luer lock cap were compared using the hypothesis test (2-sample *t*-test, CI 0.95, p-value<0.05).

### Phase 2/Hygiene and patient safety

In the 2nd phase of the project, cultures were carried out at the TWC C-ports to evaluate possible contamination and thus health risks for patients. The choice which of the TWC C-ports was provided with the luer access split septum system (Group A) or the conventional luer lock cap (Group B) was by chance. Before intervention the luer access split septum system connected to the C-port has been cleaned with a wiping disinfection using an antiseptic pad, the luer lock cap connected to the C-port has been cleaned with a spraying disinfection.

All used TWC were from adult patients treated in the Department of Radiooncology, University Hospital Regensburg, Germany. All patients were asked in front to give their written informed consent. The research was in compliance with the Helsinki Declaration (http://www.wma.net/en/30publications/10policies/b3/index.html.pdf). An ethical committee approval for the study was not necessary, because no human material and personal data was used.

### Culture material and microbiology

Directly after the change of a TWC, cultures were taken of the interior of the C-port using a sterile cotton carrier (TRANS system 110 c with Amies-gel agar without coal, hain lifescience). The criterion for exclusion was previous administration of antibiotics.

For the culture, a sterile cotton carrier was laid onto the ground of the C-port under sterile conditions. The cotton carrier was then pressed at the lateral wall and the base of the housing with rotating motions to reach the entire inner surface of the system.

For maintaining sample quality, the procedure was specified by means of a standard operating procedure. The cotton carriers were kept in a transport medium and immediately taken to a microbiological laboratory (Department of Medical Microbiology and Hygiene, University Hospital Regensburg, Regensburg, Germany). The samples were then planted onto Columbia agar with 5% sheep blood (Oxoid) and McConkey agar, incubated for 48 hours at 36 +/- 2°C, and checked for growth. A sample was stated as positive with the presence of one or more colony forming units (CFU). In case of a positive result (evaluated as *positive* sample), the bacteria were specified by means of biochemical examination.

Samples without any signs of bacterial growth after 48 hours (no CFU) were defined as sterile and evaluated as *negative* sample. We examined 50 samples of both systems in accordance with the described protocol. The 100 samples were taken from 25 patients. 12 patients belonged to group A (4 samples each patient), 12 patients to group B (4 samples each patient). Only one patient (two times for admission) belonged to both groups (2 samples for each study group).

The 24h routine changing period of TWC’s remained unaltered in the program, so that the average intervention rate was 4 interventions during one routine changing period.

## Results

### Phase 1/Economics and work simplification

The efficiency of the modified approach was tested and calculated by means of 1000 discrete event simulation runs [7]. With the new access system, the mean working time of 5.5 minutes could be reduced to 2.97 minutes.

The difference of the values was caused by the compartmentalized handling of the luer lock caps (design, packaging, location) as well as by the partial work intensification and “repeating rate”, for instance, by unintentional dropping or premature contamination of luer lock caps.

The process instability of luer lock caps as one-time usable articles and the related inefficiency of the actual process are shown in Figure 3.

**Figure 5:**
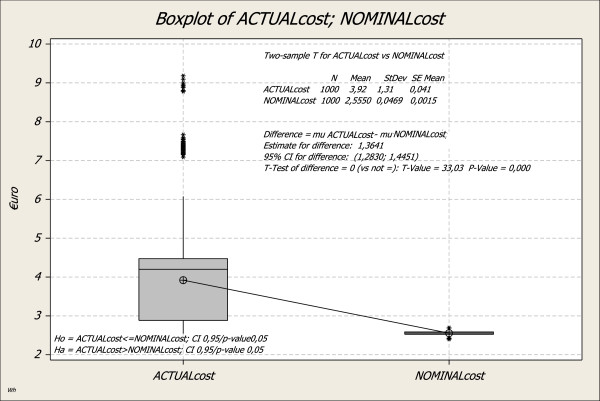
**2-sample-t/process costs: The box (-plot) represents the mean 50% of the values, the upper and lower orthogonal line (called whisker) the 25% of the superior and lower values.** The line drawn through the box demonstrates the median values. The outliers are displayed with asterisks. The line between the ACTUALcost and NOMINALcost links the mean values, the symbols in the charts stand for the mean value of all samples, which serves as an estimate for m.

For calculating average process costs (labour time multiplied with salary and material costs per use), we used 1000 simulations resulting in 3.92 € for luer lock caps and 2.55 € for the luer access split septum system. The hypothesis test (Figure 5) (2-sample *t*-test, CI 0.95, p-value<0.05) confirmed the significance of the result, which is very positive for hospitals.

### Amortization of material expenses

**Figure 6:**
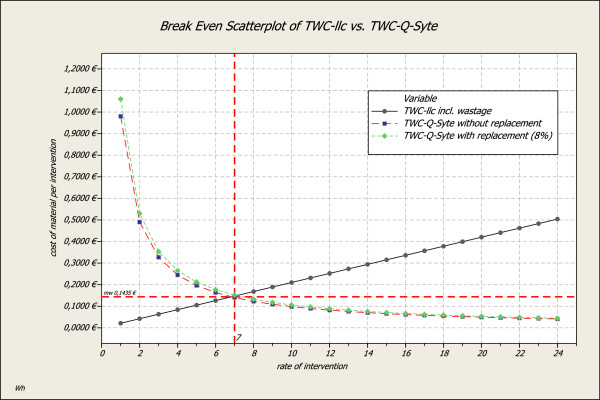
Amortization of material expenses per intervention: TWC with luer lock cap (TWC-llc) vs. TWC with BD Q-Syte® (TWC- BD Q-Syte®).

When comparing the mere non-personnel cost proportion per intervention and work process, a non-personnel cost-referred amortization of the BD Q-Syte® access system can be achieved after 6 to 8 interventions during the routine TWC change periodicity – despite the higher initial costs of the BD Q-Syte® access system (Figure 6).

Figure 6 shows that non-personnel costs for a one-time usable article increase linearly with multiple usage, whereas these costs decrease per intervention for the luer access split septum system as a re-usable article.

This difference is mainly caused by the material property of one-time usable and re-usable articles.

Luer lock caps as one-time usable articles have to be discarded and replaced for every intervention (mathematical: adding) compared to the luer access split septum system as a re-usable article that remains at the tree-way cock (mathematical: division).

### Phase 2/Hygiene and patient safety

In phase 2, we examined the question of improved hygiene and risk minimization for a possible transmission of infection to the patient. In 50 reviewed samples (TWC’s), the contamination rate for the luer lock cap was 8% (4 out of 50 samples were positive).

In addition to the expected presence of typical skin bacteria (especially S. epidermidis) in low (2 samples) and high number (1 sample), we also detected a large number of mixed gram-negative flora in a pure culture, i.e. Escherichia coli, Klebsiella oxytoca, and Acinetobacter lwoffii. This result was most likely caused by a contamination of the TWC with bacteria of intestinal flora, whose pathogenic potential is significantly higher than of S. epidermidis.

In contrast, the contamination rate of the 50 samples (TWC’s) with the luer access split septum system was 0% (no bacterial growth was found in any of the 50 samples).

Additionally, possible hygienic risks (related to material, surroundings, and staff handling) could be reduced by 65.38% based on the discrete events in comparison of the ACTUAL work process (Figure 3) with the NOMINAL work process (Figure 4).

## Discussion

When new consumable products are introduced into clinical routine, appropriate methods are often lacking for showing possible advantages or disadvantages in a scientific reviewable manner. If such new materials are also associated with higher costs, the work process should be first analyzed in detail. In this way, possible benefits for staff and patients can be determined, and macroeconomic decisions can be taken. The importance of such “process analyses” for all processes in a hospital is also increasingly reflected in the literature [6,8].

So far, new consumable products have been introduced in hospitals for a defined time period at which end the “test phase” was compared with the “pre-situation”. This process is time-consuming and often not reliable enough, because staff members have to familiarize themselves with the new product only for a short period of time, which often results in motivation problems that may negatively affect final results.

A simulation compiled by a few employees only, who become “integrated” into the process, creates a reliable description of work processes and work analysis. This description can be converted into a software-supported simulation that can be run so frequently that it corresponds to actual conditions.

Apart from saving time, the simulation also helps to analyze own operational sequences with regard to possible optimizations, as such simulations are necessary for the introduction of standard operation procedures (SOP) in the context of quality management.

An analysis of the work process with regard to the intervention at the middle port (C-port) of a TWC followed by a simulation of 1000 interventions should first show if working time for staff could be reduced with the new consumable product. Secondly, the analysis should determine the intervention threshold from which possible higher initial costs of a new product in comparison to a conventional system are justified as well as other possible advantages.

By measuring the contamination level of the two products, we could not only show cost efficiency benefitting hospitals but also benefits for patients. Such analyses should first and foremost focus on patient safety. In their paper “Scrub the Hub” on infection due to contamination of central venous catheters (CVC), Lockman et al. [9] stressed that interventions carried out according to high hygienic standard is the foremost prerequisite for avoiding CVC infections. The cleaning intensity as well as the technical condition of the connecting material before and after the intervention is crucial. The poorer the cleaning, the less accessible or more complex the material, the poorer is the quality of disinfection. In daily clinical routine especially, work intensification may result in neglecting the essential waiting period after C-port disinfection.

A retrospective multi-center study by Hetem et al. [10] showed a high rate of contamination of central venous catheters at different connection sites with Staphylococcus aureus; up to 12% of patients suffered from bacteremia after CVC removal. The contamination rate indicated in the trial of Casey et al. [11] with a contamination rate of 10% for the internal surfaces of TWC luers with standard caps correspond with the data obtained in our observation. It might be objected that our observation was not a prospective, randomized trial and reports only experience at a single institution.

Above all, it is remarkable that the contamination of the TWC’s was already detectable after a very short retention time. The presence of gram-negative bacteria indicates that contamination is not only caused by typical skin flora but also by bacteria with a higher pathogenic potential.

The impact of using needle-free connecting systems for nosocomial infections has been examined in several trials. Salgado et al. [12] could reduce a high level of bacteremia after changing Split septum systems (similar to the luer access split septum system used here) to systems with mechanical flaps (5.95 vs. 1.79 per 1000 catheter days, RR 3.32, p<0.001). In a multi-center trial, Jarvis et al. [13] also found a significant increase of the rate of bacteremia when using systems with mechanical flaps compared with Split septum systems or needles (9.49 vs. 6.15 per 1000 CVC days, RR 1.54, p<0.001). Additionally, the authors could also show the benefit of Split septum systems, as in the second phase of the trial a change of systems significantly decreased infection rates (9.49 vs. 5.77 per 1,000 CVC days, RR 1.65, p<0.001). Basically, a central venous catheter is a risk factor [14] for hospital-associated bacteremia; therefore, every intervention of a catheter system necessitates a high level of hygiene.

## Conclusions

In the present research, the luer access split septum system tested in this observation with a divided split septum was superior to conventional luer lock caps. The advantage of the luer access split septum system lies in the simplified handling for staff, which results in a reduced risk of patient infection due to improved clinical hygiene. Thus, the higher price of the luer access split septum system we used compared to conventional luer lock caps is being put into perspective when considering the high intervention rates, for example, in intensive care units, or the complex intravenous infusion schedules, such as in cytostatic drug protocols.

## Competing interests

***Potential conflicts of interest***. WH is employed at the Risk Management, Becton Dickinson GmbH, Heidelberg, Germany. He was in an advisory capacity for the discrete event simulation. Becton Dickinson GmbH, Heidelberg, Germany paid also for the cultures and hygienic analyses. All other authors: no conflicts.

## Authors’ contributions

FP, WH and TH designed the observation and wrote the observation protocol. SG, WH and TH collected the data, OK and MGH supervised the overall conduct of the observation. FP, WH and TH did the analysis, FP wrote the first draft of the paper. All authors were involved with the interpretation, critical review of the paper, and gave final approval of the manuscript.

## Pre-publication history

The pre-publication history for this paper can be accessed here:

http://www.biomedcentral.com/1471-2334/14/41/prepub
